# Psychogenic Polydipsia Presenting as Recurrent Hyponatremic Seizures in a Patient With Schizoaffective Disorder

**DOI:** 10.7759/cureus.96407

**Published:** 2025-11-09

**Authors:** Roli R Gupta, Gouthami S G, Shivakumar Chaukimath, Santosh Ramdurg, Manovijay B Kalasagond

**Affiliations:** 1 Department of Psychiatry, Shri. B. M. Patil Medical College, Hospital and Research Centre, BLDE Deemed to Be University, Vijayapura, IND

**Keywords:** antipsychotic treatment, behavioral therapy, electroconvulsive therapy, fluid restriction, hyponatremia, psychiatric comorbidity, psychogenic polydipsia, schizoaffective disorder, seizures, water intoxication

## Abstract

Psychogenic polydipsia is a rare but potentially fatal condition characterized by excessive water consumption leading to dilutional hyponatremia and neurological complications. It is most frequently seen in patients with chronic psychotic disorders such as schizophrenia and schizoaffective disorder. We present a 42-year-old man with a 22-year history of schizoaffective disorder who developed recurrent generalized tonic-clonic seizures and confusion. Initial evaluation revealed severe hyponatremia, but the cause could not be identified. He was admitted to an intensive care unit and treated with hypertonic saline and antiepileptic medication. After stabilization and discharge, he experienced difficulty in sleeping and hallucinatory behavior and was subsequently readmitted under psychiatric care. During routine workup, laboratory investigations confirmed hyponatremia again, which was then further investigated for causes and found. The patient had hypotonic hyponatremia with low serum osmolality and inappropriately dilute urine, consistent with psychogenic polydipsia. Endocrine and systemic causes, including diabetes insipidus, adrenal insufficiency, and hypothyroidism, were excluded. Drug history was taken in detail to rule out any drugs causing hyponatremia and substance abuse was ruled out as well. The patient was observed during his ward stay and it was found that he would drink excessive amounts of water, exceeding 15 liters daily, including water from storage tanks and washing areas. Management focused on cautious correction of serum sodium to prevent osmotic demyelination, positive and negative reinforcement for fluid restriction, seizure prophylaxis, and optimization of psychiatric treatment. He underwent nine sessions of bilateral electroconvulsive therapy for refractory affective and psychotic symptoms and participated in behavioral modification sessions aimed at reducing water-seeking behavior. Sodium levels normalized within 10 days, and no further seizures occurred. At the six-month follow-up, the patient remained stable under supervised care, compliant with medication, and maintained a restricted fluid intake below two liters daily. This case underscores the importance of early recognition of psychogenic polydipsia as a reversible cause of recurrent seizures and hyponatremia in psychiatric populations. Vigilant monitoring of fluid intake, interdisciplinary management, and behavioral intervention are crucial to achieving sustained recovery and preventing recurrence.

## Introduction

Psychogenic polydipsia is a behavioral disorder characterized by excessive water intake in the absence of physiological stimuli. It occurs in approximately six to twenty percent of patients with schizophrenia and schizoaffective disorder [1-3]. Normal serum sodium levels range between 135 and 145 mmol/L and dilutional hyponatremia defined as serum sodium below 135 mmol/L can lead to cerebral edema, seizures, coma, and death if unrecognized. Mortality in severe untreated cases has been reported between 10 and 20% due to neurological complications [4]. Despite these potentially fatal complications, psychogenic polydipsia is often overlooked because neurological and behavioral manifestations, such as agitation or confusion, are misattributed to psychiatric relapse or medication side effects [3].

The underlying pathophysiology is multifactorial. Proposed mechanisms include dopaminergic hypersensitivity, hypothalamic dysfunction, anticholinergic-induced xerostomia, and maladaptive coping responses to emotional distress [1,5,6]. Psychotropic medications that cause dry mouth may further reinforce compulsive drinking behaviors. Early detection and integrated management are therefore crucial to prevent recurrent hyponatremia and associated morbidity [7,8].

This report describes a middle-aged man with schizoaffective disorder who presented with recurrent seizures secondary to severe hyponatremia. His case highlights the diagnostic challenges, clinical course, and multidisciplinary management approach required for psychogenic polydipsia identified through close inpatient observation.

## Case presentation

A man in his early 40s with a 22-year history of schizoaffective disorder was admitted to an intensive care unit under physician care following recurrent generalized tonic-clonic seizures, agitation, and behavioral disturbance. During these episodes, he was noted to be restless, disoriented, shouting irrelevantly, attempting to remove his intravenous lines, and occasionally becoming physically aggressive toward staff during post-ictal agitation. His speech was incoherent at times, and he appeared fearful and confused, expressing misperceptions and persecutory ideas such as believing that the medical staff were trying to harm him. Initial management focused on primary epilepsy management. Hyponatremia, with serum sodium of 116 mmol/L, was detected on routine investigations, but no clear cause was yet known. Sodium correction was done, and the patient was then later discharged after a week post correction of serum sodium levels, levels being 132 mmol/L with antiepileptic medications, and was continued on olanzapine 15mg tablets for schizoaffective disorder and was called for regular follow-ups.

Two weeks after the discharge patient was brought with complaints of disturbed sleep, irritability, verbal aggression, episodes of unprovoked shouting over trivial issues and violent behavior towards family members to the psychiatric outpatient department, where he was advised in patient care. On repeating routine investigations, the patient was again found to be hyponatremic, with serum sodium levels of 121mmol/L. Further detailed history and investigations were done to find out the cause of having hyponatremia repeatedly, findings of which are mentioned in the table below. His medical, social, and family history included chronic schizoaffective disorder with multiple hospitalizations. No history of endocrine disorders was identified. Family history was unremarkable for seizures or metabolic conditions. The patient and attenders were also asked about any substance use or other drug history or abuse but nothing significant was mentioned. Around three days post-admission in the ward, the patient was kept under strict observation and was observed to engage in water-seeking behavior, drinking more than 15-16 liters of water daily, including unsafe sources such as toilet bowls and tap water if denied safe drinking water. Collateral history confirmed longstanding psychotic symptoms and episodes of compulsive drinking that had not previously been linked to seizures. Over the course of his illness, the patient had received multiple pharmacological trials. He was initially treated with risperidone and olanzapine with partial improvement, followed by a trial of clozapine up to 300 mg/day between February 2022 and February 2023, with minimal response. Subsequent regimens included aripiprazole long-acting injection, divalproate, chlorpromazine, and sulpiride at different times to manage affective and psychotic symptoms, with limited benefit. During the current admission, he was restarted on olanzapine 15 mg/day and divalproate 1 g/day, which showed gradual improvement in mood and behavioral symptoms. The patient’s laboratory findings were consistent with primary polydipsia and hypo-osmolar hyponatremia, as summarized in Table 1.

**Table 1:** Summary of Laboratory and Radiological Investigations Demonstrating Severe Hypotonic Hyponatremia and Exclusion of Endocrine or Structural Causes. WNL: Within normal limits; mmol/L: millimoles per liter; mOsm/kg: milliosmoles per kilogram; µg/dL: micrograms per deciliter; HbA1c: glycated hemoglobin. MRI brain-Punctate T2/FLAIR hyperintense foci in the right parietal lobe, likely non-specific white matter changes; there was no acute lesion

Investigation	Result	Reference Range/Remarks
Complete hemogram	Within normal limits	—
Serum sodium (Na⁺)	116 mmol/L (on admission); 124 mmol/L (48 hours later); 132 mmol/L (day seven); 121 mmol/L (on readmission)	135–145 mmol/L
Serum potassium (K⁺)	4.2 mmol/L	3.5–5.1 mmol/L
Serum urea	24 mg/dL	15–45 mg/dL
Serum creatinine	1.0 mg/dL	0.8–1.3 mg/dL
Serum osmolality	252 mOsm/kg	275–295 mOsm/kg
Urine osmolality	120 mOsm/kg	300–900 mOsm/kg
Urine sodium (Na⁺)	<20 mmol/L	Low, suggestive of dilutional hyponatremia
Thyroid function test	Within normal limits	Excluded hypothyroidism
Serum cortisol	12.8 µg/dL	4.3–22.4 µg/dL; excludes adrenal insufficiency
Antidiuretic hormone (ADH)	1.1 pg/mL	Low; excludes SIADH
Blood glucose (fasting)	72 mg/dL	<100 mg/dL
Blood glucose (postprandial)	120 mg/dL	<140 mg/dL
HbA1c	5.30%	<5.7%
Electroencephalography	Awake interictal EEG is normal; correlates clinically.	---
CT scan head	Nil significant finding	—
Echocardiography	Nil significant finding	Excluded cardiac cause
Ultrasonography (whole abdomen)	nil significant finding	Excluded hepatic and renal causes

The behavioral formulation suggested that his water intake provided transient relief from dysphoria, anxiety, and xerostomia, thus being reinforced despite repeated harm.

The patient was placed under strict fluid restriction and continuous observation. Behavioral interventions, including psychoeducation and positive reinforcement for limiting water intake, were initiated. Considering the persistence of psychotic and affective symptoms despite pharmacotherapy and the behavioral component of compulsive water drinking, a course of bilateral modified electroconvulsive therapy (ECT) was planned. The patient received nine bilateral modified ECT sessions on alternate days over three weeks under anesthesia and muscle relaxation. ECT was well tolerated, and gradual improvement in mood, behavioral control, and psychotic symptoms was observed after the fourth session. His fluid-seeking behavior reduced significantly thereafter, and serum sodium stabilized to 138 mmol/L. Adjustments to psychiatric medications were made to optimize psychotic symptom control and was started on tablet divalproate 500 milligrams twice daily along with tablet olanzapine fifteen milligrams. Attenders were educated about the hazards of excess water consumption by the patient and were trained to keep him under observation and also about reinforcement techniques. Over the course of hospitalization, serum sodium normalized, and the patient exhibited improved behavioral control. He was discharged with a detailed care plan emphasizing fluid restriction, close monitoring, and psychiatric follow-up. The family and patient were explained about danger signs such as confusion, hypersomnia, unusual behavior, persistent headache, vomiting, muscle weakness, seizures, or unresponsiveness, and asked to seek immediate medical help if any of these occur.

## Discussion

Psychogenic polydipsia is a rare but potentially life-threatening condition, most commonly affecting patients with chronic psychotic disorders such as schizophrenia and schizoaffective disorder. Its prevalence among psychiatric patients ranges from six to twenty percent [1-3]. The condition is frequently under-recognized because the resulting hyponatremia and seizures may mimic primary neurological disorders or psychiatric exacerbations [3,4]. This case highlights that compulsive water intake can remain hidden, and hyponatremia may be the only clue, emphasizing that awareness of psychogenic polydipsia should extend beyond psychiatry to other specialties, including internal medicine, neurology, and critical care.

The pathophysiology of psychogenic polydipsia is multifactorial. Dysregulated dopaminergic signaling may enhance thirst, while hypothalamic dysfunction and anticholinergic-induced xerostomia from psychotropic medications contribute to excessive water intake [1,5,6]. Compulsive drinking may also serve as a maladaptive coping mechanism to relieve anxiety, dysphoria, or negative affect, creating a self-reinforcing cycle of behavior despite physiologic harm [6]. Differentiating psychogenic polydipsia from other causes of hypotonic hyponatremia, including syndrome of inappropriate antidiuretic hormone secretion, diabetes insipidus, adrenal insufficiency, and thyroid dysfunction, is essential to guide appropriate management and prevent complications [7,9].

Management requires a multidisciplinary approach. Acute hyponatremia should be corrected gradually with careful monitoring to avoid osmotic demyelination [9]. Long-term strategies include strict fluid restriction, behavioral interventions such as scheduled and supervised water access, and psychoeducation for patients and caregivers [6,10]. In refractory cases, pharmacologic agents such as clozapine or vasopressin receptor antagonists may be considered, although evidence remains limited [10]. ECT may be particularly beneficial in patients with treatment-resistant affective or psychotic symptoms, as illustrated in this case.

In the present case, the patient had previously received multiple pharmacological trials, including risperidone, olanzapine, aripiprazole, sulpiride, and chlorpromazine, with partial or transient response. A supervised trial of clozapine up to 300 mg/day over one year (February 2022 - February 2023) resulted in minimal improvement in both affective and psychotic symptoms. Considering the limited response and subsequent tolerability challenges, clozapine was discontinued, and treatment was optimized with olanzapine 15 mg/day and divalproate 1 g/day during the current admission. This combination was selected for its synergistic efficacy in schizoaffective disorder, targeting both mood stabilization and psychotic symptom control while minimizing the risk of further hyponatremia or seizure precipitation.

This report underscores that psychogenic polydipsia is an under-noticed cause of hyponatremia and seizures, and vigilance is necessary not only in psychiatry but across other specialties where unexplained hyponatremia may present. Early recognition and coordinated management combining medical stabilization, psychiatric optimization, behavioral supervision, and caregiver education can prevent life-threatening complications. Clinicians should maintain a high index of suspicion for psychogenic polydipsia in psychiatric patients with recurrent or unexplained hyponatremia [1-4,6-8].

## Conclusions

Psychogenic polydipsia is an under-recognized cause of severe hyponatremia that can present with seizures, often masquerading as primary neurological disorders. Its compulsive water intake may remain hidden, even in patients adherent to psychiatric treatment, making it a potentially life-threatening condition if not identified early. Awareness of psychogenic polydipsia should extend beyond psychiatry to internal medicine, neurology, and critical care specialties, as early recognition is critical for preventing complications. A multidisciplinary management approach, combining medical stabilization, psychiatric optimization, behavioral supervision, and caregiver education, is essential to ensure patient safety and long-term stability.
